# Silence of the Electrocardiogram: A Case Series With Occluded Coronaries and Normal Electrocardiograms

**DOI:** 10.14740/jmc5210

**Published:** 2026-02-02

**Authors:** Chris Sani, Asher Gorantla, Krishna Patel, Varsha Talanki, Varshitha T. Panduranga, Usaid Raqeeb, Nana Osei, Adam S. Budzikowski

**Affiliations:** a Department of Internal Medicine, SUNY Downstate Medical Center, Brooklyn, NY 11203, USA; b Division of Cardiology, SUNY Downstate Medical Center, Brooklyn, NY 11203, USA; c Emergency Medicine, One Brooklyn Health, Brooklyn, NY, USA; d College of Medicine, SUNY Downstate Health Sciences Center, Brooklyn, NY 11203, USA; e Division of Cardiology, NYC Health + Hospitals/Kings County, Brooklyn, NY, USA

**Keywords:** NSTEMI, Acute coronary syndrome, Revascularization, Percutaneous coronary intervention

## Abstract

Every year over a million patients present with acute coronary events. A substantial number of patients present with non–ST-segment elevated myocardial ischemia (NSTEMI), and a subset of those have normal-looking electrocardiograms (EKG). We report three cases of patients that had near-to-complete occlusions of coronary arteries with an initial nondiagnostic EKG. Silent EKG findings in the setting of coronary occlusion represent a significant challenge that may delay time to reperfusion. Our cases indicate that EKG alone may underestimate the severity of ischemia, particularly in the lateral and posterior territories. This emphasizes the importance of adjunctive tools such as ultrasound and cardiac biomarker assessment. Further research must be done to determine the risk of myocardial ischemia (MI) in normal EKG, including research in minor EKG changes, and further algorithms that could identify risk in patients in nondiagnostic EKG.

## Introduction

Every year nearly 1.2 million patients are admitted to the hospital for acute coronary events [1]. Despite advances in the early recognition and management of acute coronary syndrome, timely identification of high-risk myocardial ischemia (MI) remains challenging, especially in patients without diagnostic electrocardiographic findings. The widespread use of high-sensitivity cardiac troponin assays has improved detection of myocardial injury. However, the 12-lead electrocardiogram (EKG) continues to play a central role in triaging regarding emergent coronary angiography. Thirty percent of these patients are found to have ST-segment elevated myocardial ischemia (STEMI) for which immediate recanalization is attempted. However, in the other 70% of patients who present with NSTEMI, intricacies of diagnosis and pathways of care often contribute to delayed treatment and reperfusion as they often have not been taken to the lab for 24–48 h [2]. Coronary angiographic studies have shown that a substantial portion of NSTEMI patients have acute and persistent total occlusion of a culprit coronary artery, often in the left circumflex artery (LCX) or its branches [3]. These STEMI equivalents often go unnoticed, and current guideline-directed strategies such as the TIMI score may inadequately identify patients. By presenting these three cases, where there were minimal EKG changes, we highlight the deceptive nature of seemingly benign EKGs in the setting of acute coronary occlusion and underscore the need for heightened clinical suspicion and need for better assessments and tools to prevent delays in reperfusion and adverse cardiovascular outcomes.

## Case Reports

### Case 1

**Figure 1:**
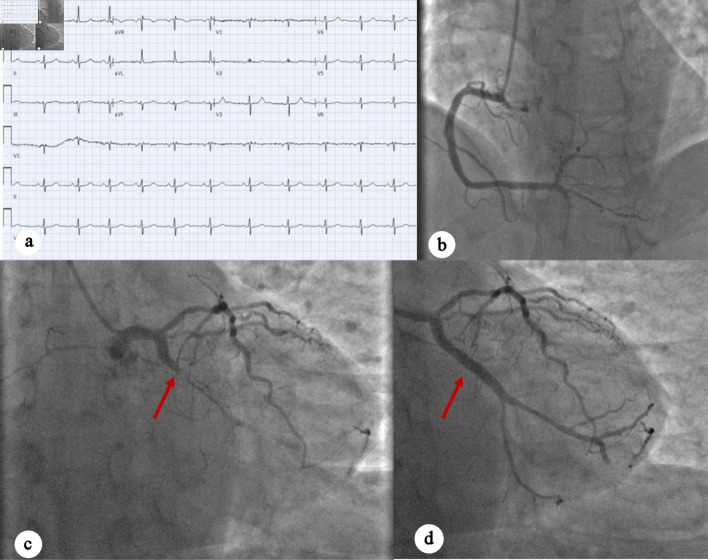
(a) EKG showing sinus rhythm with no acute ischemic changes. (b) Left anterior oblique caudal view showing right coronary artery. (c) Left anterior oblique caudal view showing complete occlusion of left circumflex artery (red arrow). (d) Post-PCI perfusion of the left circumflex artery (red arrow). EKG: electrocardiogram; PCI: percutaneous coronary intervention.

A 51-year-old man with a history of prediabetes, asthma, and obstructive sleep apnea presented with intermittent burning chest pain lasting 2 weeks. The pain was exertional, worsened with emotional distress, radiated to his throat and left scapula, and improved with rest. The patient’s family history is significant for MI in his father at age 67. The social history revealed recent occupation-related stress, with no history of substance use disorder. On the day of admission, his symptoms did not improve with rest or omeprazole. In the emergency department (ED), the patient was hemodynamically stable, and troponins were elevated at 0.6 ng/mL to 1.96 ng/mL (normal < 0.10 ng/mL). EKG showed sinus rhythm at a heart rate of 60 beats per minute without clear ischemic changes (Fig. 1a). Cardiology was consulted due to elevated troponin. Bedside point-of-care ultrasonography (POCUS) showed no overt regional wall motion abnormalities; however, due to the concerning clinical presentation and rising troponin levels, the interventional cardiology (IC) team was activated. The patient underwent left heart catheterization (LHC), which showed three-vessel obstructive coronary artery disease (CAD) with a proximal LCX total/subtotal thrombotic occlusion. Percutaneous coronary intervention (PCI) with placement of a drug-eluting stent (DES) was successfully performed (Fig. 1b–d). Left ventricular (LV) systolic function was preserved, with a low LV end-diastolic pressure of 5 mm Hg. The patient was admitted to the cardiac critical care unit (CCU) for monitoring, and transthoracic echocardiogram (TTE) the following day revealed normal LV size with preserved ejection fraction (EF) of 55–60%, mild inferolateral hypokinesis, and normal diastolic function. Trace mitral regurgitation was noted. Troponin was trended till downtrend, and the patient was discharged after 12 days with a follow-up with IC team.

### Case 2

**Figure 2:**
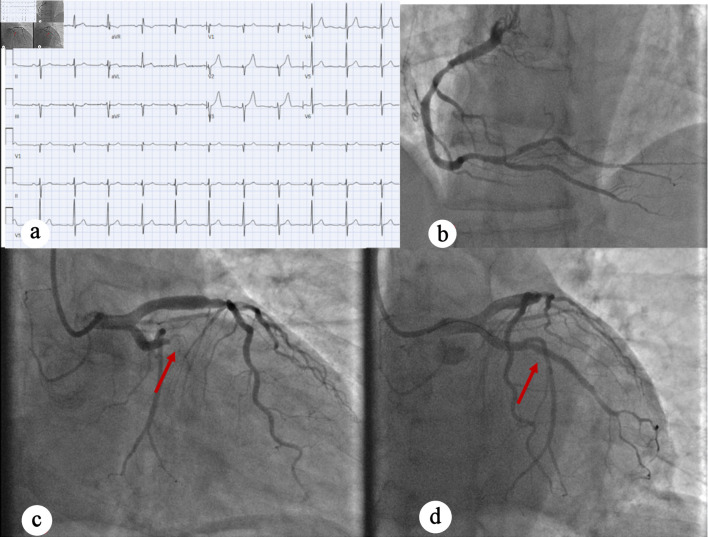
(a) EKG showing normal sinus rhythm with no acute ischemic changes. (b) Left anterior oblique caudal view showing early-mid-RCA with moderate nonobstructive disease. (c) Left anterior oblique caudal view indicating complete occlusion of OM 100% occlusion, as well as mid-LAD moderate disease (red arrow). (d) Left anterior oblique caudal view showing post-PCI perfusion of OM1 (red arrow). EKG: electrocardiogram; RCA: right coronary artery; LAD: left anterior descending artery; OM: obtuse marginal artery; PCI: percutaneous coronary intervention.

A 63-year-old male with a history of type 2 diabetes mellitus, hyperlipidemia, and insulinoma status post resection in 2000, presented to the ED with acute-onset abdominal pain that started the night before and persisted into the morning. In the ED, patient blood pressure was 150/94 mm Hg, computed tomography (CT) angiography of the chest and abdomen did not show any acute vascular or aortic pathologies. An EKG was done and initially interpreted as normal sinus rhythm with left anterior fascicular block (Fig. 2a). Cardiology was consulted for elevated troponin I to 13.72 ng/mL. On cardiology evaluation, POCUS showed a reduced EF and decreased wall motion of the inferior lateral wall on an apical view of the heart, which was confirmed on parasternal short-axis view. The IC team was promptly activated, and the patient underwent LHC, which showed a 100% proximal obtuse marginal artery occlusion with successful placement of a DES (Fig. 2b–d). The patient also had 60-70% moderate stenosis of the mid left anterior descending artery (LAD), with an instantaneous wave-free ratio suggesting the lesion was physiologically nonsignificant, and 50% moderate stenosis of the mid right coronary artery. Left ventriculography showed mildly reduced EF with anterior and lateral hypokinesis. The patient was transferred to CCU, where a formal TTE was done the next day, which showed normalized LV systolic function without regional wall motion abnormalities. The patient was discharged with follow-up.

### Case 3

**Figure 3:**
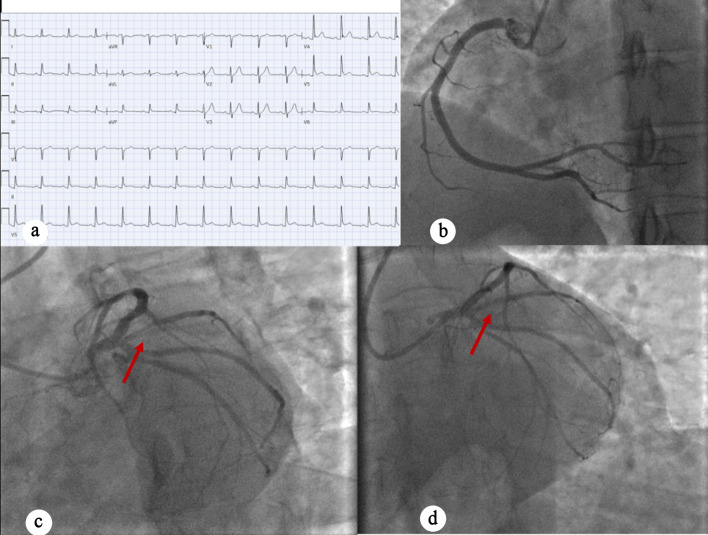
(a) EKG showing normal sinus rhythm with no ischemic changes. (b) Right anterior oblique caudal view showing normal RCA. (c) Coronary angiography indicating 90% proximal ramus intermedius stenosis (red arrow). (d) Left anterior oblique caudal view post-PCI perfusion of ramus intermedius (red arrow). EKG: electrocardiogram; RCA: right coronary artery; LAD: left anterior descending artery; OM: obtuse marginal artery; PCI: percutaneous coronary intervention.

A 45-year-old man with no significant past medical history presented with chest discomfort, low-grade fever, body aches, and fatigue for 3 days. The chest pain was left-sided and radiated to his back. He denied palpitations, orthopnea, paroxysmal nocturnal dyspnea, abdominal pain, nausea, or vomiting. The patient’s family history is significant for myocardial infarction in his father at age 53. In the ED, he was hypotensive (96/62 mm Hg), but otherwise hemodynamically stable. Labs were notable for elevated troponins at 4.45 ng/mL. EKG again was nondiagnostic for ischemia (Fig. 3a). Cardiology was consulted for NSTEMI, and the patient was loaded with aspirin, ticagrelor and intravenous heparin. He was admitted to the CCU for further management. TTE showed normal LV size and wall thickness with preserved systolic function (EF 55-60%), no regional wall motion abnormalities, grade I diastolic dysfunction, and trace mitral regurgitation. As the patient continued to experience chest pain, the IC team was activated. LHC showed single-vessel CAD with 90% stenosis of the proximal Ramus intermedius, which was treated with DES (Fig. 3b–d). During the procedure, transient occlusion of the LAD and LCX occurred, with suspected secondary air embolism versus thrombus that resolved with heparinized saline. Intraoperatively, the patient developed ventricular tachycardia and new ST elevations, which required a rapid response evaluation. A second rapid response was called for acute hypoxic respiratory failure with concern for flash pulmonary edema in the setting of elevated left-sided pressures requiring intubation and mechanical ventilation. The patient was noted to be hypotensive and required initiation of norepinephrine and dopamine for cardiogenic shock. An intra-aortic balloon pump (IABP) was placed through the right femoral artery with 1:1 augmentation. The patient was transferred to the CCU with gradual improvement. He was extubated, vasopressors and oxygen requirements were down-titrated, and the IABP was removed. TTE performed 2 days post-LHC revealed normal LV size and wall thickness with preserved systolic function (EF 70-71%), no regional wall motion abnormalities, grade I diastolic dysfunction, and trace mitral and tricuspid regurgitation. Troponin levels initially trended upward during hospitalization and were followed until a downward trend was observed. The patient was hemodynamically stable and was discharged after 11 days with follow-up.

## Discussion

In the era of high-sensitivity troponins, understanding EKGs is key in understanding the complexities of coronary ischemia in the emergency setting. However, even EKG can be deceptive in the presence of significant ischemic injury. In our three cases, our patients presented with varying presentations of chest pain, elevated cardiac biomarkers, but grossly unremarkable EKGs. While subtle abnormalities were presents, there were no diagnostic EKG findings suggestive of near-complete or complete occlusion of coronary arteries, especially in the lateral distribution of the left ventricle, including the LCX, obtuse marginal branches of the LAD, or the ramus intermedius. Although certain clinical and ancillary diagnostic clues prompted early activation of the IC team, without a high index of suspicion and the use of adjunctive testing, such as POCUS , these cases could have been overlooked, leading to delays in appropriate reperfusion therapy

While NSTEMI is generally attributed to incomplete, transient, or dynamic coronary thrombosis, these patients demonstrated an acute and persistent occlusion of a culprit coronary artery [4]. Current NSTEMI guidelines only suggest immediate invasive strategy in only a subgroup of patients. These include unstable or very high-risk patients including patients with hemodynamic instability, cardiogenic shock, acute heart failure, refractory angina, or electrical instability. Other high-risk patients for which LHC can be done include patients with a GRACE score greater than 140, diabetes, age > 75, or ongoing dynamic ST-segment changes [4]. Another is a continuing steep rise in cardiac biomarkers which can indicate ongoing ischemia and myonecrosis despite optimal therapy.

EKG changes such as ST-segment depressions and acute T wave inversions are common findings in NSTEMI patients. However, 12-lead EKG has decreased sensitivity in the inferior, lateral and posterior myocardial walls due to lead placement constraints [5]. As in our patients, where the occluded arteries supplied these territories, a STEMI-equivalent presentation was effectively concealed by nondiagnostic EKG findings. Other studies have shown that silent EKGs in STEMI equivalents are often seen with RCA occlusion [6]. Early use of posterior leads in patients with typical symptoms may be beneficial. There are other EKG findings including Wellens syndrome, de-Winter St/T-wave complex, N-wave, and T-wave precordial instability, which have been shown to be STEMI equivalents in NSTEMI patients [7]. However, subsequent validation studies have yielded mixed results, limiting their routine clinical application [4].

Patients presenting with silent EKG findings tend to be younger to middle-aged and are more likely to be male, as seen in our patients [2]. There are significant subsets of NSTEMI patients with underlying total occlusion of a culprit artery (TOCA) that cannot be reliably identified using contemporary risk stratification tools, such as the GRACE or TIMI scores [2]. NSTEMI patients with TOCA thus present an immense practical challenge. A 2021 study demonstrated that, compared to NSTEMI patients without TOCA and STEMI patients, NSTEMI patients with TOCA were much more likely to undergo cardiac arrest during angiography [8]. Another study described that patients with NSTEMI and TOCA of the LCX were more likely to suffer from cardiac arrest and had higher mortality compared to STEMI [9]. These findings underscore the importance of maintaining clinical vigilance and avoiding premature exclusion of life-threatening coronary disease solely based on EKGs in patients with chest pain.

There are still many limitations in the field of IC and emergency medicine that could delay care in the setting of NSTEMI. Although risk stratification tools such as the GRACE, TIMI, and HEART scores assist operators in determining the time of LHC, delays in definitive management remain common. Artificial intelligence–based algorithms, such as those used in the ROMIAE multicenter study, have shown some benefits in the early diagnosis of ischemia in the emergency setting [10]. However, there is still work left to be done to make emergency personnel more aware of the risks of trusting a “silent” EKG and to promote a more cautious, multimodal diagnostic approach in patients presenting with suspect acute coronary syndrome.

### Conclusions

Patients with elevated troponins may not always receive the degree of clinical vigilance warranted, particularly when electrocardiographic findings are normal or nondiagnostic. In our case series, three patients with nonischemic EKGs were found to have significant coronary artery occlusions, where delayed care could have led to worse cardiovascular outcomes, as illustrated by the complicated clinical course of our third patient. Elevated troponin levels with nondiagnostic EKGs do not rule out complete occlusions, especially in the lateral distribution of the heart or the RCA. Careful monitoring of EKGs, troponin trends, clinical presentation/history, and POCUS is needed to facilitate early identification of high-risk ischemia. A normal or nondiagnostic EKG in the setting of elevated troponin should therefore not provide false reassurance or preclude timely invasive evaluation. Further studies need to be done to better triage patients in the setting of chest pain and elevated troponin. Further studies are needed to refine triage strategies and improve early detection of high-risk coronary occlusions, as current diagnostic algorithms and risk stratifications tools do not reliably identify patients with life-threatening coronary disease, particularly those with silent EKG findings.

## Data Availability

Any inquiries regarding supporting data availability of this study should be directed to the corresponding author.
